# Prolactin-Responsive Circular RNA circHIPK3 Promotes Proliferation of Mammary Epithelial Cells from Dairy Cow

**DOI:** 10.3390/genes11030336

**Published:** 2020-03-20

**Authors:** Jia Liu, MoLan Zhang, DeWei Li, MengLu Li, LingHao Kong, MengWen Cao, YanHong Wang, ChengChuang Song, XingTang Fang, Hong Chen, Hu Xu, ChunLei Zhang

**Affiliations:** 1 Institute of Cellular and Molecular Biology, School of Life Science, Jiangsu Normal University, Xuzhou 221116, Jiangsu, China; lj15152181726@163.com (J.L.); 15052013859@163.com (M.Z.); wwwlideweicom@163.com (D.L.); m15705213732_1@163.com (M.L.); 18260358609@163.com (L.K.); CAOmengWEN525@163.com (M.C.); 7096871@163.com (Y.W.); chengchuangsong@163.com (C.S.); chenhong1212@126.com (H.C.); 2 Kunming Police Dog Base of the Ministry of Public Security, Kunming 650204, Yunnan, China; ahutiger2001@tom.com

**Keywords:** PRL, circHIPK3, STAT5, splicing factors, lactation regulation

## Abstract

The highly expressed circHIPK3 is a circular RNA that has been previously reported to regulate the growth of human cells. In this study, we found an increased expression of circHIPK3 in bovine mammary epithelial cells treated with prolactin (PRL) in high-throughput sequencing data. Thus, we further investigated the effect of circHIPK3 on the proliferation and differentiation of mammary epithelial cells. We used qRT-PCR/Cell Counting Kit-8 (CCK-8) and a Western blotting analysis to evaluate the effects on cell proliferation. We found that circHIPK3 promotes the proliferation of mammary epithelial cells. The STAT5 signaling pathway was previously associated with the prolactin response and when the STAT5 was suppressed, the expression of circHIPK3 decreased. The results suggest that the response to prolactin and the associated STAT5 signaling pathway affect the expression of circHIPK3, which subsequently affects the proliferation of mammary epithelial cells in dairy cows.

## 1. Introduction

Lactation persistency (LP) influences the overall milk production of dairy cattle and animal health, making it an economically important trait for dairy cattle. The number and activity of milk-secreting cells determines the persistency of lactation [1]. Prolactin (PRL) plays a key role during pregnancy and the postpartum development of the mammary gland for successful lactation [2]. PRL is not only a key regulator of the mammalian reproductive process but is also involved in the process of breast proliferation and differentiation during pregnancy [3,4]. Chronic administration of the PRL-release inhibitor quinagolide can suppress cell proliferation and enhance apoptosis in the mammary tissue of lactating dairy cows [5], whereas PRL injections lead to a higher cell proliferation rate. Besides, treated with quinagolide injection can reduce the apoptosis of mammary epithelial cells in lactating dairy cows [6]. PRL acts to stimulate the proliferation of bovine mammary gland epithelial cells [7]. PRL can activate *STAT5a* and *STAT5b* through JAK2 in the epithelium [8,9] to regulate the specification and proliferation of alveolar progenitors as well as the survival of their functionally differentiated mammary gland epithelial cells [10]. In addition, Elf5 (through the JAK−STAT−Elf5 pathway) also plays an important role in this process [11]. However, PRL specific molecular mechanisms regulating the proliferation of mammary epithelial cells are unknown.

Circular RNA (circRNA) is a class of RNA whose members have 3′ and 5′ ends that are joined together via exon or intron circularization. Recently, circRNA have been demonstrated to be abundant, stable, conserved and nonrandom [12,13]. Emerging evidence has shown that some circRNAs may have potentially important roles in gene regulation, including the initiation and elongation of RNAP II-transcribed genes, linear splicing, the stability of microRNA-regulated linear mRNAs, translation, and ribosome biogenesis [12,13].

We previously detected the expression of thousands of circular RNAs in the mammary glands of a cow and a rat, with circHIPK3 present at a high abundance [14,15]. Interestingly, circHIPK3 has been reported to promote the proliferation of tumor cells, such as HuH-7, HCT-116, HeLa, PC-3, and DU145 cells [16,17,18]. Additionally, circHIPK3 can promote the proliferation and differentiation of chicken myoblast cells [19]. The goal of this work was to test whether PRL regulates the proliferation of mammary epithelial cells through circHIPK3. Additionally, the role of the STAT5 signaling pathway in the effect of PRL on circular RNA was investigated in cattle mammary epithelial cell lines.

## 2. Materials and Methods

### 2.1. Animals and Cell Lines

Healthy mastitis-free Holstein cows from the Xuzhou Lvjian Dairy Farm (Xuzhou, China) and C57BL/6J mice purchased from Xuzhou Medical College (n ≥ 3, each) were selected for this study. Heart, liver, spleen, lung, kidney, stomach, intestine, and mammary gland samples were collected and preserved in −80 °C refrigerator. The mouse mammary epithelial cell line (HC11) was obtained from the ATCC (Manassas, VA). The dairy cow mammary epithelial cell line (MAC-T) was a gift from Dr. Lili Zhao of Northwest A&F University (Xi’an, China). The animal experiments were approved by the Ethical Committee on Animal Care and Use of Jiangsu Normal University of Xuzhou, China (20190509).

### 2.2. Cell Culture

MAC-T cells were cultured in growth medium (Dulbecco’s modified Eagle’s medium (DMEM) supplemented with 10% fetal bovine serum, 10 µg/mL of insulin, 100 IU/mL penicillin, 100 µg/mL streptomycin) at 37 °C in 5% CO_2_ for 4 d. The cells were cultured (5 × 10^4^ cells/well in 6-well plate) in serum-free DMEM for 16 h. And then the control groups were cultured in a growth medium which only contained with hydrocortisone (1 µg/mL) for 72 h the PRL groups were cultured in an added 1 µg/mL HC and 5 µg/mL ovine PRL growth medium for 72 h. The medium was changed daily and the total cellular mRNAs were extracted.

HC11 cells were grown in a RPMI-1640 medium with 10% fetal bovine serum, 5 μg/mL insulin and 10 ng/mL EGF (Epidermal Growth Factor). For the PRL treatment, cells at 100% confluence were grown for 16 h in a medium without EGF supplementation, followed by growth in a RPMI-1640 medium supplemented with 1 μg/mL HC, 5 μg/mL insulin and 5 μg/mL PRL for 72 h. For the transient transfection, HC11 cells were transfected using Lipofectamine 2000 (Invitrogen, Carlsbad, CA, USA) according to the manufacturer’s protocol. CircHIPK3 siRNA and non-targeting control siRNA were purchased from GenePharma (Shanghai, China). The siRNA sequences are listed in Supplementary Table S1.

For STAT5 inhibition, the cells were treated with or without 50 µM STAT5 inhibitor (nonpeptidic nicotinoyl hydrazine compound CAS 285986-31-4; Merck Millipore, Billerica, MA) or DMSO (Merck Millipore) as the vehicle control for 1 h before the PRL treatment.

### 2.3. RNA Library Preparation

Total RNA samples were treated with RNase R (Epicentre, Madison, WI, USA) at 37 °C for 15 min (3 U RNase R/1 μg RNA). Next, cDNA library preparation was performed from the RNase R-treated RNA samples (500 ng/μL) using the Illumina TruSeq RNA Sample Preparation Kit v2 (Illumina, San Diego, CA, USA) following the manufacturer’s instructions. The libraries were sequenced on the Illumina HiSeq 2500 platform with 2 × 150 bp paired-end reads.

The details of the bioinformatics analysis, including the sequencing data processing, the transcript assembly, and the circRNA identification, were described previously [15].

### 2.4. Real-Time PCR

Complementary DNA (cDNA) was synthesized using random primers and the reverse transcription kit PrimeScript RT Master Mix (Takara, Dalian, China) from the total RNA. A quantitative real-time PCR (qRT-PCR) analysis was carried out using the SYBR Premix Ex TaqTM kit (TaKaRa, Dalian, China) on a StepOne Plus Real-Time PCR System (Applied Biosystems, Carlsbad, CA, USA). The detected differences of circRNA were normalized to the levels of GAPDH or U6. The primer sequences are listed in Supplementary Table S1.

### 2.5. Western Blotting Analysis

Cellular proteins were isolated by incubating cell monolayers with an RIPA protein extraction reagent (Beyotime, Beijing, China) supplemented with a protease inhibitor cocktail PMSF (Solarbio; China). Aliquots (20 μg protein) of cell lysate were electrophoretically separated and then transferred to a nitrocellulose membrane. The membrane was blocked for 1 h in PBS in 10% skim milk, and then incubated overnight with primary antibodies against CDK1, Cyclin A2 (HuaBio, Cambridge, MA, USA), and GAPDH (AbSci, Baltimore, MD, USA). After a final wash, the blots were incubated with their respective secondary horseradish peroxidase-conjugated antibodies. Visualization of peroxidase positive bands was performed using an enhanced chemiluminescence (ECL) reagents (Bio-Rad, Hercules, CA, USA) and imaged by FluorChem M (Alpha, San Jose, CA, USA). Signal intensities of the proteins were quantified with Alpha View software and normalized to the signal of GAPDH.

### 2.6. Cell Proliferation Assay

The proliferation of MAC-T and HC11 cells was detected using the Cell Counting Kit-8 (CCK-8). Cultured cells under different treatments were collected 24 h and 48 h after treatment. After washing with PBS, these cells were incubated with 100 μg/ml CCK-8 (EnoGene, Nanjing, China) for 1 h at 37 °C. The cells were then detected by 450 nm absorbance using a microplate reader (Thermo Scientific, Waltham, MA, USA). Data were obtained from three independent experiments.

### 2.7. Statistical Analysis

A statistical analysis of the results of Western blotting and real-time PCR were performed using GraphPad Prism version 6.0 (GraphPad Software, La Jolla, CA, USA). All the data are presented as the mean ± SEM from at least three independent experiments. Statistical significance was considered at a value of *p* ≤ 0.05.

## 3. Results

### 3.1. Length Source Distribution and the Differential Expression of circRNAs

The CIRI algorithm [20] is an annotation-independent approach for circRNA detection and was used here to identify circRNA transcripts. In total, 7924 circRNA transcripts were expressed across the two treatment groups (Table S2). The edgeR software package (Development version, Bioconductor, Buffalo, US) was used to identify 159 circRNAs as differentially expressed between the control and PRL groups (*p* < 0.05) which are listed in Table S2.

**Figure 1:**
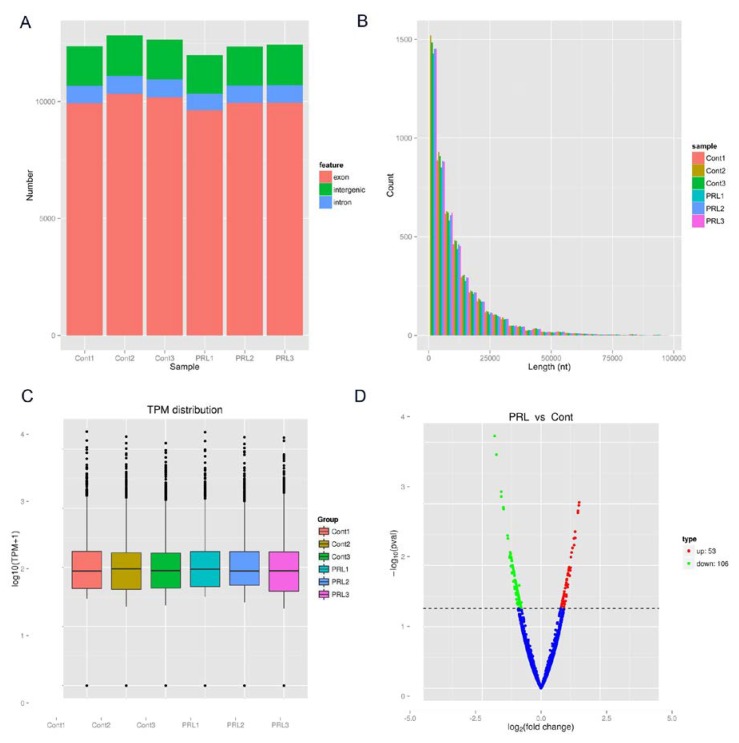
High expression of circRNAs in dairy cow mammary epithelial cell lines (MAC-T cells) after prolactin (PRL) treatment. (**A**) Genomic regional distribution of circRNAs in control and PRL-treated MAC-T cells; (**B**) Length distribution of circRNAs in control and PRL-treated MAC-T cells; (**C**) The box plot of the global expression of circRNAs in control and PRL-treated MAC-T cells; (**D**) Volcano plot constructed using fold-change values and p-values. The vertical lines correspond to 2.0-fold up- and down-regulation between control and PRL-treated MAC-T cells (control vs PRL), and the horizontal line represents a P-value. The red and green points in the plot represent the differentially expressed circRNAs with statistical significance. TPM reads transcripts per million.

The analysis of the source distribution of circRNAs indicates that most of the circRNAs are derived from exons in the PRL-treated and PRL-untreated samples, with only a few circRNAs from introns or intergenic regions (Figure 1A). The length analysis shows that the number of detected circRNAs is inversely proportional to the corresponding length, so that the longer the length, the smaller the number of circRNAs. Thus, the possibility of circRNAs generation is higher when the length of circRNAs is less than 2000 nt, and this possibility decreases gradually with the increase of length (Figure 1B). The expression level of circRNAs is normalized as Transcripts Per Million (TPM) to determine the specific expression of each circRNA in each sample with or without PRL treatment (Figure 1C). The differential expression analysis revealed 53 up-regulated circRNAs and 106 down-regulated circRNAs in MAC-T cells treated with PRL (Figure 1D). The results suggest that there are a large number of circRNAs in the control and PRL groups, and that their formation depends on the moderate length of exons or intron sequences. Meanwhile, some circRNAs might play an important role during lactation in the milk gland epithelial cells of dairy cows.

### 3.2. Identification of circHIPK3 as a Candidate circRNA

**Figure 2:**
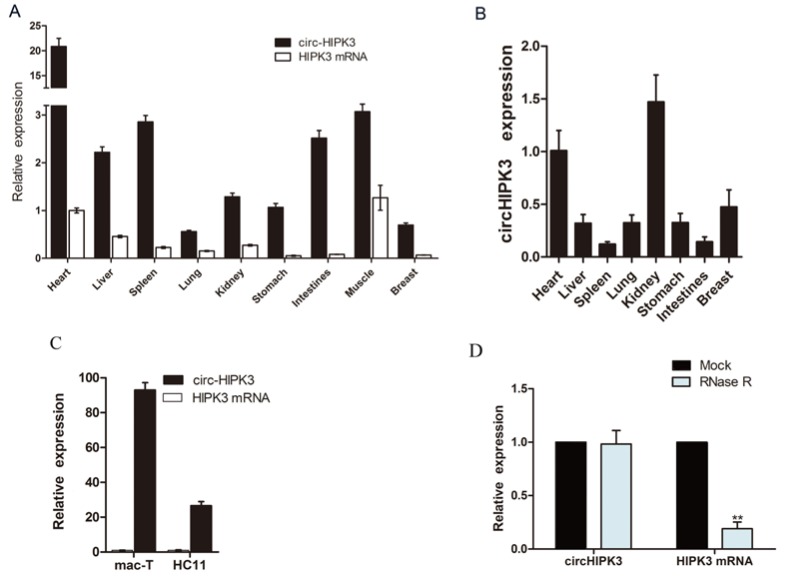
The circHIPK3 is widely expressed. (**A**) The expression profile of circHIPK3 and HIPK3 mRNA in cattle tissues; (**B**) The expression profile of circHIPK3 and HIPK3 mRNA in mouse tissues; the mean ± SEM of tissues from six cow or mouse samples; (**C**) The expression profile of circHIPK3 and HIPK3 mRNA in MAC-T cells and mouse mammary epithelial cell lines (HC11 cells). The mean ± SEM of three samples and three independent experiments; (**D**) The expression of circHIPK3 and HIPK3 mRNA after treatment with RNase R. Mean ± SEM of three samples from three independent experiments. * *p* < 0.05; ** *p* < 0.01.

Twenty candidate circRNAs exhibited a significant differential expression between the control and PRL groups and were selected for further research (Table S2). Of these, only 10 circRNAs contained reverse splicing sites (Figure S1). A BLAST analysis was used and detected a sequence identical between the circHIPK3 sequence and the published bovine genome sequence (NM_001193007.3). According to the qRT-PCR analysis, there was a significantly greater abundance of circHIPK3 in cattle tissues than there was of the HIPK3 mRNA (Figure 2A). However, the situation of the abundance of circHIPK3 and HIPK3 mRNA was the opposite in mouse tissues (Figure 2B). The circHIPK3 was relatively stable in the breast tissues of both species. There was a relatively higher amount of circHIPK3 in dairy cow breast epithelial cells (Figure 2C), which significantly increased under prolactin treatment in MAC-T cells (Figure S2A). The same result was detected in the HC11 cell line (Figure S2B). RNase R treatment was used to confirm the circular structure of circHIPK3. The relative expression of circHIPK3 did not change significantly after RNase R treatment (Figure 2D), which indicates that circHIPK3 was insensitive to RNase R, consistent with a circular form.

### 3.3. CircHIPK3 Promotes the Proliferation of HC11 Cells

**Figure 3:**
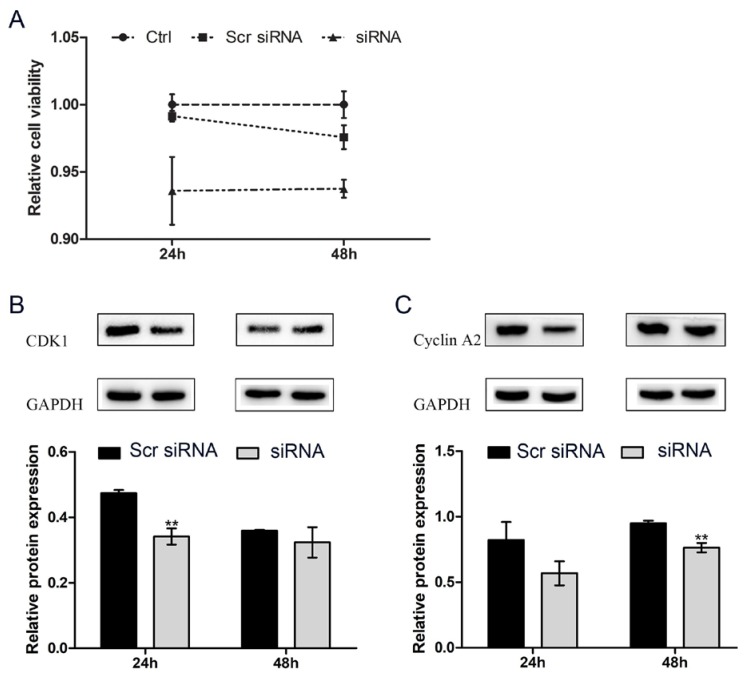
CircHIPK3 knockdown represses the proliferation of HC11 cells. (**A**) Relative cell viability of HC11 measured by CCK-8; (**B**) CDK1 protein expression; (**C**) Cyclin A2 protein expression. Mean ± SEM of three samples from three independent experiments. * *p* < 0.05; ** *p* < 0.01.

To assess the effect of circHIPK3 on proliferation, we knocked down circHIPK3 in HC11 cells by transfection with specific siRNA against circHIPK3 and then used the CCK-8 and Western blotting assays to analyze the effect of circHIPK3 knockdown on the proliferation of HC11 cells. Because general liposomes can hardly be transferred into MAC-T cells, we only explored the effect of circHIPK3 on HC11 cells. The CCK-8 assay show that the reduced expression of circHIPK3 markedly suppressed the proliferation of HC11 cells (Figure 3A). CDK1 [20] and Cyclin A2 [21] can be used as proliferation marker genes of the mammalian cell cycle. Western blotting showed that circHIPK3 knockdown reduced the protein expression levels of both CDK1 and Cyclin A2 (Figure 3B,C). Overall, these results suggest that circHIPK3 promotes the proliferation of HC11 cells.

### 3.4. The STAT Pathway Promotes the Proliferation of Mammary Epithelial Cells

**Figure 4:**
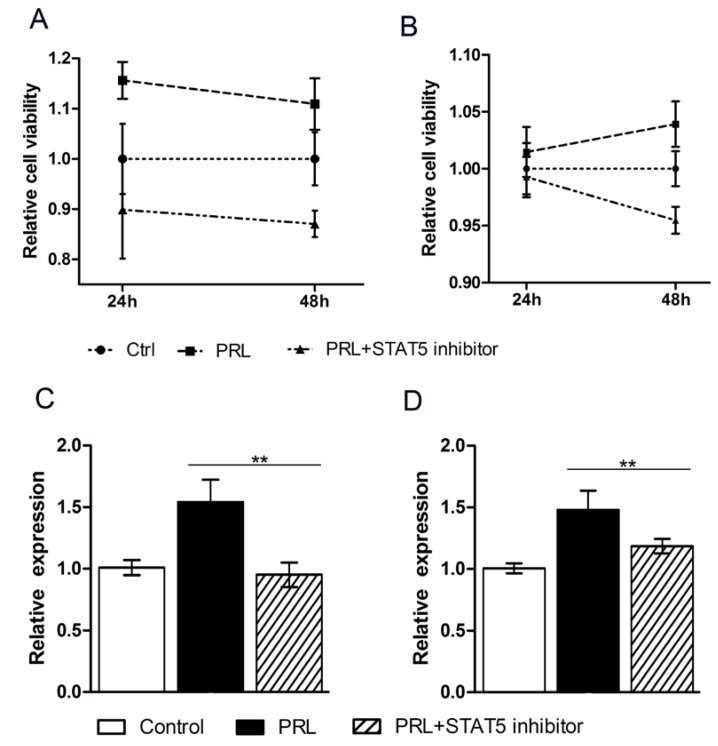
The STAT5 knockdown inhibits the expression of circHIPK3. (**A**) The relative cell viability of MAC-T cells; (**B**) The relative cell viability of HC11 cells; (**C**) The circHIPK3 expression in MAC-T inhibited by the STAT5 inhibitor; (**D**) The circHIPK3 expression in HC11 cells inhibited by the STAT5 inhibitor. Mean ± SEM of three samples from three independent experiments. * *p* < 0.05; ** *p* < 0.01.

To explore the regulatory mechanism of PRL and a potential role of the STAT pathway, a STAT5 inhibitor was transfected into the cell culture medium. The CCK8 analysis revealed that the cell viability of MAC-T was increased by the PRL treatment but repressed by the STAT5 inhibitor (Figure 4A); similar results were observed for mouse mammary epithelial cells (Figure 4B). The expression of circHIPK3 is closely related to the STAT5 signaling pathway, which is induced by prolactin in MAC-T (Figure 4C) and HC11 cells (Figure 4D). Compared to the untreated group, there was a significant improvement of circHIPK3 expression with PRL treatment, which was restored to a normal level when the STAT pathway was inhibited.

### 3.5. PRL Affects circHIPK3 Expression and Regulates Alternative Splicing Factors

As described above, we found that circHIPK3 can affect the proliferation of HC11 cells. However, the regulation mechanism was unclear. We then analyzed the levels of alternative splicing factors that are related to the STAT5 signaling pathway induced by prolactin. The increased expression of some protein kinases may affect the formation of alternative splicing processes in mammals [22]. Thus, we explored whether the expression levels of these alternative splicing factors would change with PRL and PRL+STAT inhibitor treatment. Prolactin increased the expression of SRPK1, SRPK2, ADAR, and ILF3, and a STAT inhibitor repressed the expression of these genes (Figure S3). In addition, the expression of the above splicing factors decreased when treated with PRL and the STAT inhibitor compared with the protein expression level in the group only treated with prolactin. These results suggest that prolactin can affect the expression of the above splicing factors through the STAT pathway, thereby changing circHIPK3 expression in mammary gland cells. However, the details of this regulation will need to be further investigated.

## 4. Discussion

Previous studies have identified thousands of circular RNAs in mammalian cells [23], which are derived from exons, introns, intergenic and UTR regions. In general, most circRNAs arise from exons, and intergenic transcripts tend to be expressed at low levels [24,25]. This is consistent with our experimental results. Most circRNAs are more than 200 nt and diverse in length [26]. In this work, for transcriptions less than 2000 nt in length, it was more likely to produce a circular structure, and there was an inverse proportional relationship within a certain range between length and cyclization rate.

The circHIPK3 is derived from exon2 of the HIPK3 gene and is an important circRNA involved in the proliferation and differentiation of several kinds of human cells [16]. Most studies of circHIPK3 have focused on tumor cells. The expression of circHIPK3 can promote the proliferation, migration and invasion of colorectal cancer cells by sponging miR-7 [16], and promote the growth of gallbladder cancer cells [27] and lung cancer cells [28] by sponging miR-124. Additionally, circHIPK3 can promote the proliferation and differentiation of chicken myoblast cells [19], and inhibit the apoptosis of human osteoblasts [29]. These results led us to question whether circHIPK3 plays an important role in the mammary gland. We detected the expression of circHIPK3 in cow and mouse tissues and mammary epithelial cell lines, suggesting that circHIPK3 was conserved. Additionally, we detected a tissue-specific variation in its expression. The expression of circHIPK3 is significantly higher than that of the associated linear RNA isoform in cattle tissues, which is consistent with a previous study of the expression of circHIPK3 and HIPK3 mRNA in human tissues [19]. This may suggest that circHIPK3 has important functions. In this study, we confirmed that circHIPK3 can promote the proliferation of mouse mammary epithelial cells. However, its specific mechanism of action is not clear. In many studies, circular RNA has been shown to act as a miRNA sponge, so we looked at miRNA involved in lactation regulation. It has been reported that miR-103, miR-486 and many other miRNAs regulate breast development and lactation [30,31,32]. We hypothesized that circHIPK3 may bind to specific miRNAs to affect breast development. To investigate this possibility, we used the RegRNA 2.0 software ((Molecular Bioinformatics Center, National Chiao Tung University, Taiwan, China) to identify miRNAs that could be potential targets for sponging by circHIPK3. The analysis identified several miRNAs with the potential for binding, including miR-134-5p, miR-191-3p, miR-143-5p, miR-667-5p, and miR-1249-5p. One of the identified miRNAs, miR-134-5p, was reported in rats to exhibit a significantly down-regulated expression after seven days of lactation compared with the expression level on the first day of lactation [33]. Another miRNA, miR-143-5p, is down-regulated in breast cancer [34]. Thus, miR-134-5p and miR-143-5p have the potential to be bound by circHIPK3 and have functions related to breast function and lactation, so these should be further investigated as targets of circHIPK3 that potentially explain its role in breast development or diseases.

An additional hint as to the function of circHIPK3 is that its expression increased after prolactin treatment. Prolactin (PRL) is a protein hormone secreted by the anterior pituitary cells, and acts in animal mammary gland development and milk production. The Jak-STAT signaling pathway is an essential pathway for PRL induction [35]. PRL can stimulate the STAT5 pathway to affect cell proliferation and differentiation [36]. In this study, we used the STAT5 inhibitor to verify the effect of prolactin on the expression of circHIPK3. The expression of circHIPK3 was detected by qRT-PCR in mouse epithelial cells treated with prolactin or prolactin plus the STAT5 inhibitor. The results show a significantly decreased expression of circHIPK3 in cells treated with prolactin and the STAT5 inhibitor, which verifies that prolactin could significantly increase the expression of circHIPK3. The expression of circHIPK3 is regulated by prolactin, but the mechanism is not clear.

We further investigated the variable splicing influencing factors that are closely related to the STAT5 signaling pathway. Alternative splicing is a common phenomenon in mammal cells [37]. The splicing effect of CD44 can be regulated by modifying Sam68 in the MAPK signaling pathway. The activated AKT signaling pathway directly acts on the SR protein or indirectly transmits a signal to the nucleus through a special kinase of the SR protein, such as SRPK2 or Clk/Sty [38]. In addition to the AKT signal pathway, the JAK/STAT, ERK signal pathways can also act in splicing. SRPK1 and SRPK2 kinases belong to the SR protein kinase family and can activate the SR protein [22]. The expression of alternative splicing factors induced by the signal pathway is closely related to the formation of circRNAs. Multiple NF90 (Nuclear Factor 90) isoforms can be produced by the immune response factor ILF3 in a viral infection, which are involved in the synthesis of circRNAs [39]. These splicing factors may be related to circHIPK3 expression and function. The results showed an association of the expression levels of SRPK1/SRPK2/ADAR and ILF3 with the STAT5 signaling pathway. There was an increased expression of these splicing factors with PRL treatment, which also increased the expression of circHIPK3. Based on this finding and the previous association of these factors with the STAT5 signaling pathway, circHIPK3 in mouse mammary gland cells may be regulated by SRPK1/SRPK2/ADAR and ILF3 in the STAT5 signaling pathway induced by prolactin. However, the detailed mechanism by which prolactin regulates circHIPK3 circulation remains unknown, and further work is required to determine how this circRNA contributes to the complex regulation of lactation.

There is a clear effect of circHIPK3 on the proliferation and differentiation of mammary epithelial cells. Because circRNA can act to sponge miRNA, future work should explore the relationship network between circRNA and miRNA and the downstream target genes of miRNA.

## Supplementary Materials

The following are available online at https://www.mdpi.com/2073-4425/11/3/336/s1, Figure S1: The structure diagram of splicing sites, Figure S2: Relative expression of circHIPK3 and HIPK3 mRNA under PRL treatment in mammary gland cells, Figure S3: PRL affects expression of alternative splicing factors, Table S1: Primer sequence information, Table S2: Relative differentially expressed circRNA transcripts between the control and PRL groups.
