# Supplementary material for: Prolactin-Responsive Circular RNA circHIPK3 Promotes Proliferation of Mammary Epithelial Cells from Dairy Cow
Source: Genes (Basel). 2020 Mar 20;11(3):336. doi: 10.3390/genes11030336 (PMC7141114; doi:10.3390/genes11030336)
Supplement: Supplementary file 1 [file genes-11-00336-s001.zip › supplementary materials/Table S1.docx]

**Table S1 :**

Table S1 Primer sequence information

| Primer name | Sequence name（5’to 3’） |
| --- | --- |
| bta-cirANKS3-F | CCCTGGATCTTCACACAGCG |
| bta-cirANKS3-R | GGGTCGGTTAGGTCTTTCCT |
| bta-cirATF6-F | TGAAGCGGCAACTGGATG |
| bta-cirATF6-R | GCGGGACTGACAAGCTGACT |
| bta-cirDEK-F | TCAGTTCAGTGGCTTTCC |
| bta-cirDEK-R | TGTTCCTGGTCTGTTGTA |
| bta-cirECH1-F | GCTATGAGTCCCTTCGGGTG |
| bta-cirECH1-R | ATTGGGCGAAGGTTGAGACC |
| bta-cirH2AFY-F | TCCCAGAAGAAGCCCGTGTC |
| bta-cirH2AFY-R | CCCACTGGCTATGGTGACTCCT |
| bta-cirHERC4-F | CTCTGGACTGGACAGCAGTG |
| bta-cirHERC4-R | TGCTCCTCTGCTCCTCTCTT |
| bta-cirHIPK3-F | TGTTCGACAGCCATACAGGG |
| bta-cirHIPK3-R | TACGGTGGGTAGACCAAGACT |
| bta-cirMRC2-F | CACAACAGCCTTGCCTTACATC |
| bta-cirMRC2-R | GGCCATCATGTACACGCACTT |
| bta-cirSAMD4A-F | ACTCATCTTCATCCGTCCCC |
| bta-cirSAMD4A-R | ACTTTTGCGTCGAGGTTTCC |
| bta-cirHIPK2-F | AGGTCTTATCCATGCCGACC |
| bta-cirHIPK2-R | TCTTCACGCTACAGAAGGCA |
| bta-HIPK3-F | GCGACACATGGTAAACCCAC |
| bta-HIPK3-R | TGCTGTGTCCTACCAGACCA |
| mmu-cirHIPK3-F | GGATCGGCCAGTCATGTATC |
| mmu-cirHIPK3-R | ACCGCTTGGCTCTACTTTGA |
| mmu-HIPK3-F | GTGATCCGGCCTGTTCTTCA |
| mmu-HIPK3-R | TGACTGGCCGATCCAAAGTC |
| mmu-Actin-F | GGCACCACACCTTCTACAATG |
| mmu-Actin-R | GGGGTGTTGAAGGTCTCAAAC |
| mmu-cdk1-F | ACGGCTTGGATTTGCTCTCA |
| mmu-cdk1-R | ACGATCTTCCCCTACGACCA |
| mmu-cyclin A2-F | GTCCTGGATTGGGTCACTGG |
| mmu-cyclin A2-R | ATGGGAGCGTTAGGACCTCT |
| bta-SRPK1-F | CAGAAGTGGCTACGGGACTG |
| bta-SRPK1-R | AACGGCTGGCCAGTTATTCA |
| bta-SRPK2-F | GCTGCTAGGCAGTATCCCAA |
| bta-SRPK2-R | CGCAGCTCTCCTTTTCTGGT |
| bta-ADAR-F | TCGTGACAGTTTCCAGCTCC |
| bta-ADAR-R | ACCGTCTTGGGAGTACCTGA |
| bta-ILF3-F | GTGAGAAGTCCATCGGCACA |
| bta-ILF3-R | GCTGCGCACTCTGTGTGATA |
| siRNA-circHIPK3 | GGUACUACAGGUAUGGCCU |
