# Supplementary figures and images for: Prolactin-Responsive Circular RNA circHIPK3 Promotes Proliferation of Mammary Epithelial Cells from Dairy Cow
Source: Genes (Basel). 2020 Mar 20;11(3):336. doi: 10.3390/genes11030336 (PMC7141114; doi:10.3390/genes11030336)

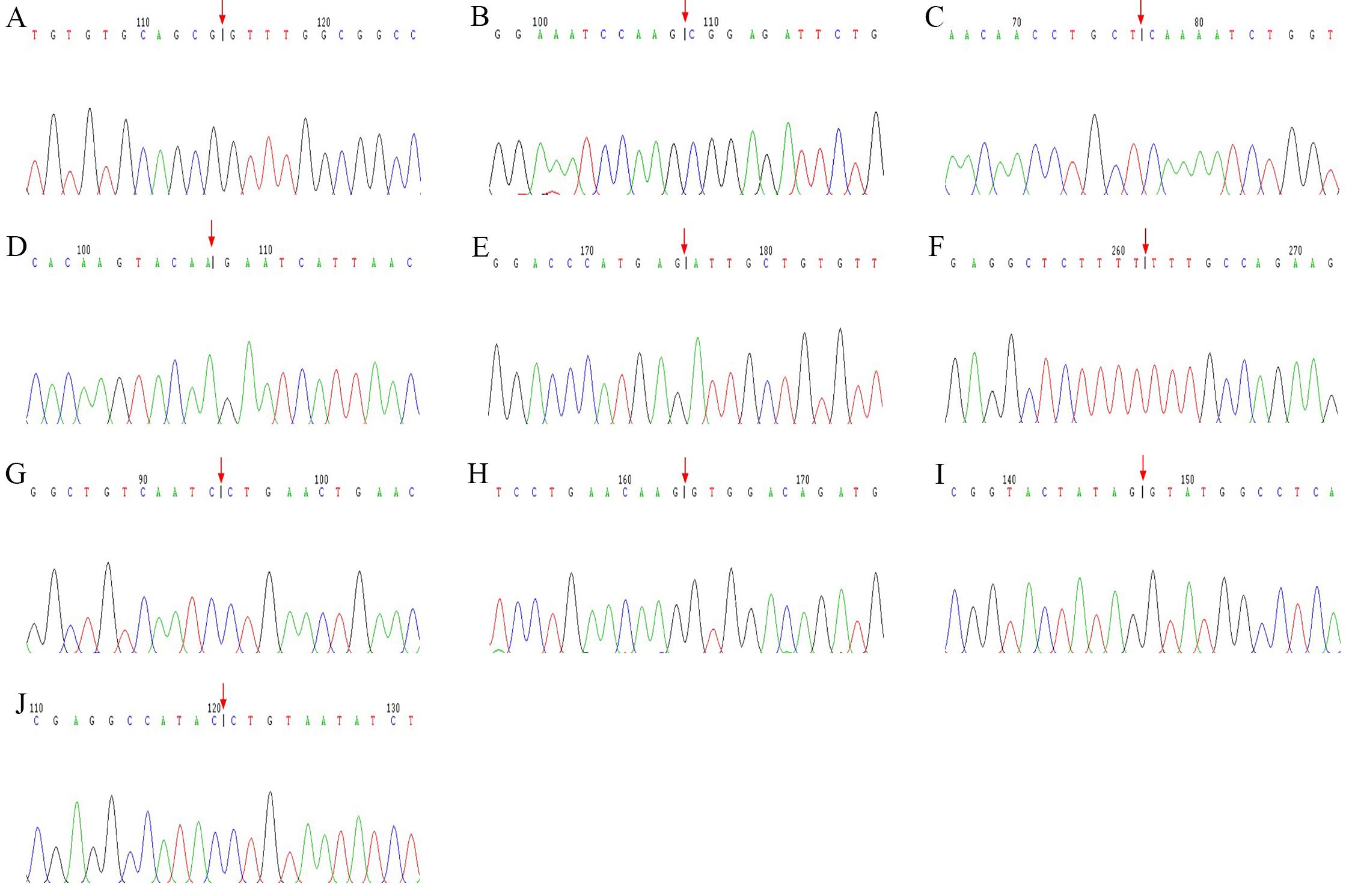

Supplement: Supplementary file 1 [file genes-11-00336-s001.zip › supplementary materials/Figure S1.jpg]

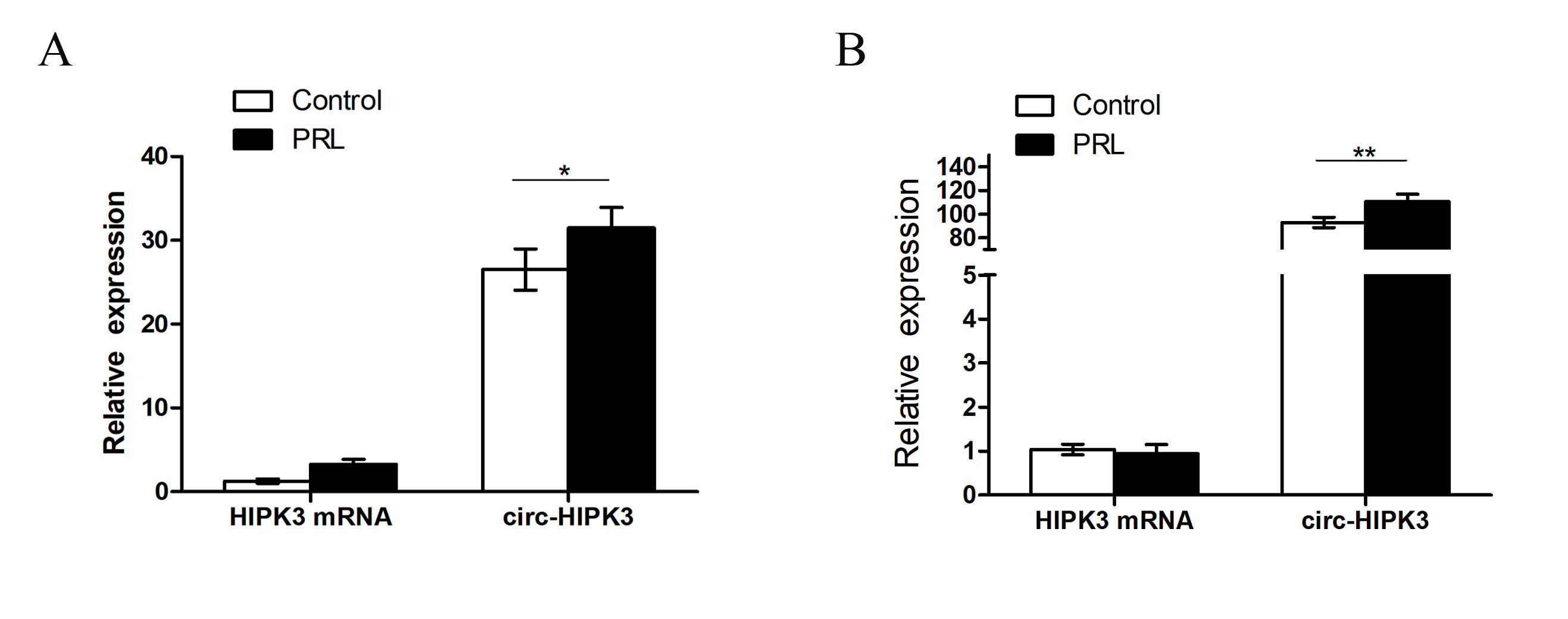

Supplement: Supplementary file 1 [file genes-11-00336-s001.zip › supplementary materials/Figure S2.jpg]

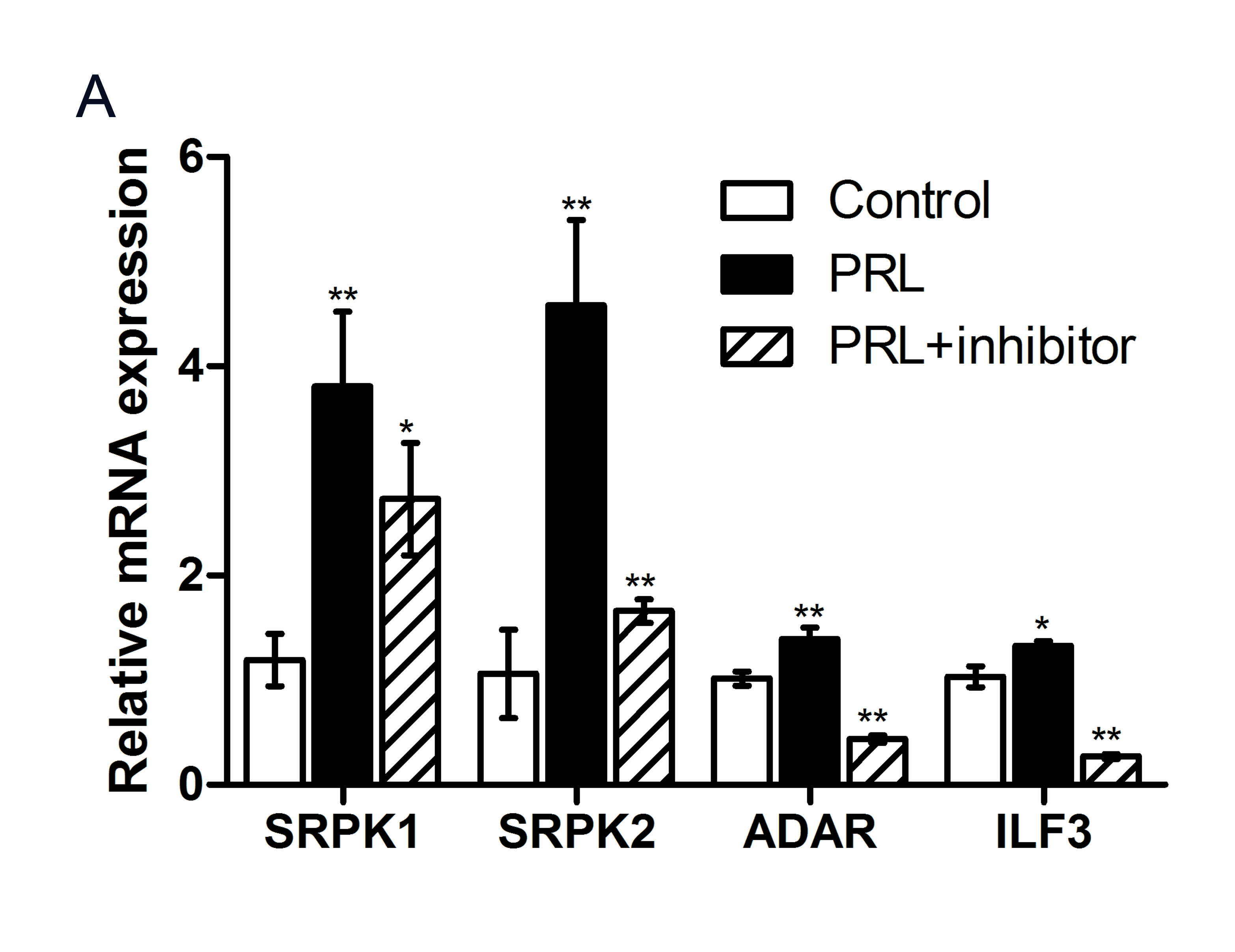

Supplement: Supplementary file 1 [file genes-11-00336-s001.zip › supplementary materials/Figure S3.jpg]
